# Margaret P. Battin, Leslie P. Francis, Jay A. Jacobson, and Charles B. Smith. Patient as Victim and Vector: Ethics and Infectious Disease, second edition: Oxford University Press; 592 pages; e-book ISBN: 9780197564547

**DOI:** 10.3325/cmj.2022.63.104

**Published:** 2022-02

**Authors:** Ana Borovečki

There are books that can be called prophetic and up-to-date whenever you read them. The book Patient as Victim and Vector: Ethics and Infectious Disease published in 2009, and republished in 2021, falls under this category.

The idea for the book emerged from a class on ethical issues in infectious diseases taught to undergraduate students. The class was held during the severe acute respiratory syndrome epidemic by three of the four authors (one a bioethics expert, one an expert in bioethics and law, and two experts in infectious diseases). However, with the advent of the SARS-CoV-2 pandemic, the authors and the publisher decided to republish the book because of its relevance and a new theoretical perspective. They were not wrong.

This is a basic book for experts working not only in epidemiology or infectious diseases, but also in disaster planning, public health, health policy, and bioethics. Moreover, this is a basic book for politicians, journalists, and everyone interested in infectious diseases, especially the coronavirus disease 2019 (COVID-19) pandemic. If this book had been read more widely, the issues that appeared in the COVID-19 pandemic would have been solved differently.

The aim of the book according to the authors was *“*to develop a general account of how best to think about contagious disease overall—not only about past outbreaks, but also about current and especially future ones.”

The book is divided into five sections. The first part, consisting of five chapters, is entitled *Seeing Infectious Diseases as Central* and represents an introduction and the outline of the book. Here, the book's approach is delineated, together with an introduction in infectious diseases for non-experts. The chapter 4 presents the advent and development of bioethics debates and explains why infectious diseases were left out of bioethics. The chapter 5 overviews the development of thinking about ethical issues in public health. Both chapters are written in a chronological and clear manner, giving one of the best and the most detailed accounts of the subject.

The second part of the book establishes the theoretical basis for the analysis of ethical issues in infectious diseases. It offers a new approach entitled “the patient as victim and vector” (PVV). This approach will be later used for analyzing different issues in other parts of the book. The approach proposes three analytical perspectives when it comes to ethical issues raised by infectious diseases:

a) The individual perspective of the patient (having or not having an infectious disease, being at risk, putting others at risk). This perspective is usually present in bioethics when analyzing ethical issues related to the physician-patient relationship. The focus is on a person, a patient with infectious disease as a victim.

b) The perspective of a community, a population with an individual as a part, a data set within this community. This perspective is typically used in public health. It is more focused on patient as a vector.

c) The perspective of theoretical uncertainty of infectious disease based on a modification of John Rawls’ “veil of ignorance.” This is “a naturalized version of the Rawlsian thought experiment from which every human being’s disease relevant situation can be seen, but in which it is not possible to know which aspects of this situation are paramount at any given point in time.” People are blinded not only to their own susceptibility to an infectious disease but also to their immunity, disease-preventing behaviors, and population they belong to. In real-life situations, not all people are equally victims and vectors. We do not know who are we going to be and to what extent. After realizing this, the third perspective makes us conclude that when it comes to infectious disease we are all in this together.

All three perspectives shift and intertwine. So, when analyzing ethical issues and developing clinical practices and research agendas in infectious diseases, one has to have in mind that a person with communicable infectious disease is both a victim and a vector.

In the third part, consisting of seven chapters, the PVV perspective is applied in different areas related to infectious diseases: traditional physician-patient relationship, research, tuberculosis treatment and isolation practices, HIV testing in pregnant women, vertical transmission of infectious diseases, antimicrobial resistance, and HPV immunization. Traditional bioethics approach to these issues is clearly differentiated from the new PVV perspective.

The fourth part, consisting of five chapters, analyzes ethical issues related to pandemic planning from the PVV perspective. This is truly a prophetic part of the book. The case analyzed is that of pandemic flu caused by H5N1 virus. However, everything presented here is relevant for the analysis of the COVID-19 pandemic response. One has a feeling that if only some of those involved in pandemic planning had gone through this section, a lot of pitfalls would have been avoided.

The final, fifth, part overviews the emerging global efforts for the control of infectious diseases. These efforts did not live up to our expectations and did not help prevent the COVID-19 pandemic. The final chapter presents the PVV perspective as a philosophical tool. In the analysis of ethical issues, PVV perspective is juxtaposed to the principalism of Tom Beuchamp and James Childress, capabilities approach of Amartya Sen and Martha Nussbaum, and liberal individualism of John Stuart Mill. PVV perspective is also used to examine public policy in the area of infectious diseases, with the analysis of real-life examples.

To summarize, the book Patient as Victim and Vector: Ethics and Infectious Disease offers a new approach to the analysis of ethical issues, clinical practices, and research agendas and policies in the area of infectious diseases with real-life and historic examples. The book has only one drawback – it brings only the US perspective, especially when analyzing the issues related to health care delivery, although sometimes comparing it with European and other contexts.

As this is the first book on the ethical issues raised by infectious diseases, it is a must read and a reference point for all the similar texts on the subject. This book is a canonical text about ethics and infectious diseases.

